# Association of stressful life events and psychological problems profile: Results from a large-scale cross-sectional study among Iranian industrial employees using Bayesian quantile structural equation model 

**DOI:** 10.17179/excli2018-1051

**Published:** 2018-07-02

**Authors:** Maryam Yazdi, Hamidreza Roohafza, Awat Feizi, Nizal Sarafzadegan

**Affiliations:** 1 Department of Biostatistics and Epidemiology, Student Research Committee, School of Health, Isfahan University of Medical Sciences, Isfahan, Iran; 2 Cardiac Rehabilitation Research Center, Cardiovascular Research Institute, Isfahan University of Medical Sciences, Isfahan, Iran; 3 Isfahan Cardiovascular Research Center, Cardiovascular Research Institute, Isfahan University of Medical Sciences, Isfahan, Iran

**Keywords:** industrial employees, psychological problems, stressful life events, quantile structural equation model

## Abstract

**Objectives**: The current study aimed at evaluating the major domains of stressful life events and their association with psychological problems profile in a large sample of Iranian industrial manufacturing employees.

**Methods**: In a cross-sectional study, 3,063 participants were randomly selected from 16,000 employees working in a big industrial company in Isfahan, Iran. Three common psychological problems i.e. depression, anxiety and psychological distress were evaluated using Persian validated version of Hospital Anxiety and Depression Scale (HADS) and 12-item General Health Questionnaire (GHQ-12), respectively. Self-perceived frequency and intensity of stressful life events were measured by stressful life event (SLE) questionnaire. Bayesian quantile structural equation model in R free statistical software (version 3.4) was used for evaluating the association of stressful life events and levels of psychological problems profile.

**Results**: Using factor analysis, two major domains i.e. socioeconomic and personal stressors were derived from 11 life stressors and a unified measure i.e. psychological problem profile was extracted from three common psychological problems. Financial and daily life stressors had the highest and sexual problems showed lowest intensity. Quantile structural equation model revealed that the psychological problems profile scores had stronger association with personal (β: ranging from 0.45, 1.87) than socioeconomics stressors (β: ranging from 0.11, 0.27). The association of socioeconomic stressors was fairly uniform across quantiles of psychological problems scores, while personal stressors showed stronger association in higher quantiles, meaning that employees with higher mental health problems more experienced personal stressors.

**Conclusions**: Life stressors particularly personal showed direct association with intensity of psychological problems in manufacturing employees. Life stressors are more perceived by employees with higher intensity of psychological problems. The results of current study can be useful in planning occupational health programs in order to improve psychological health and job productivity.

## Introduction

Psychological problems have a high prevalence among the working population that impose an enormous societal burden (Chopra, 2009[7]). Previous studies showed, in particular, common psychological problems such as depression, anxiety and psychological distress are among the most frequent causes of occupational disability (Lee, 2010[25]; Wang et al., 2003[45]).

It is well documented that stressful life events can be considered as a leading cause of psychological problems (American Psychiatric Association, 2013[2]). Stressful life events are described as discrete quantifiable circumstances that can have severe negative impacts on different aspects of life. Severe acute life events that possess a high degree of threat and unpleasantness, such as the death or illness in the family, divorce, loss of an important job, have been found consistently to precede the onset of depression (Monroe et al., 2007[29]; Paykel, 2003[37]). Chronic difficulties (e.g. health concerns, home violence, financial problems, intra and inter personal conflicts) have commonly been associated with increased risk of mood and anxiety disorders (Faravelli et al., 2007[12]; Muscatell et al., 2009[32]). Cumulative effects of daily stressors such as air pollution and traffic have been introduced as predictors of the emergence of depression and anxiety symptoms (Kioumourtzoglou et al., 2017[21]; Parrish et al., 2011[36]). Nonetheless, most of the employees-community level studies have just focused on the association of psychosocial stress at work such as effort-reward imbalance (ERI) and job strain, and ignored the importance of other sources of stress (Bonde, 2008[5]). This tendency may be due to the fact that work-related stressors are often more amenable to intervention. 

Recently some studies among workforce population have highlighted the importance of life stressors besides of work-related stressors in ameliorating employees' psychological conditions. Factors such as home life related stressors (Clark et al., 2012[8]; Cole et al., 2002[9]; Fan et al., 2015[11]; Kato and Yamazaki, 2009[19]), daily stressors and socioeconomic status (NajahAliAqe and Abdelaziz, 2017[33]; Ervasti et al., 2013[10]; Rusli et al., 2008[41]) showed significant association of mental health. These evidences highlight the importance of considering diverse sources of stress and figuring the pathways that they are associated with workforce' psychological problems.

Specific gaps in the recent studies are as follows: use of few and/or uncommon stressors, less emphasis on more prevalent, daily and chronic stressors, and a limited range of psychological outcomes. Furthermore, to our knowledge few large-scale studies have been conducted in developing countries with a focus on the psychological problems and their association with life stressors among manufacturing employees. To address these gaps, the primary objective of current study is to describe the association between a wide range of life stressors and psychological problems in a large sample of industrial manufacturing employees.

Majority of previous studies had used conventional statistical techniques that are not able to give a complete picture of the relationship between psychological problems and their related determinants. However, from a health policy viewpoint it is often important to examine how subgroups of individuals with different intensity of psychological problems are influenced by important factors such as stressful life events. On the other hand, some psychiatric research data frequently exhibit departure from normal distribution, in which mostly are empirically quite skewed; accordingly, common regression approaches might seriously under- or overestimate the significant associations or even fail to identify them (Lê Cook and Manning, 2013[23]). A robust and more complete picture of the associations could be provided by modeling quantiles of the dependent variable (whole distribution) as function of explanatory variables in a quantile regression modeling framework (Koenker, 2005[22]; Wang et al., 2015[46]). 

The objectives of the current study are 1) to investigate the most perceived and frequent stressful life events by Iranian industrial employees, 2) to classify a large variety of stressful life events in smaller domains and investigate which type of them has higher impact on employees' psychological condition, 3) to explore how different subgroups of employees with low to high level of psychological problems perceive stressful life events when adjustment is made for the impacts of demographic, job-related (e.g. work shift and ERI scale) and lifestyle-related confounding variables. The main objectives of the study were addressed through applying a comprehensive statistical modeling approach i.e. Bayesian quantiles structural equation modeling (quantile SEM) (Wang et al., 2015[46]). In the measurement part of this modeling framework because of high prevalence of comorbidity and co-occurrence of psychological problems (Wang et al., 2006[44]) a unique combined measure as psychological problems profile was extracted through combining depression, anxiety and psychological distress scores and treated as a latent response, also from eleven stressful life events two domains were extracted and were treated as latent predictors; finally in structural part of the model the quantiles of psychological problems profile were regressed on the extracted life stressors domains.

## Methods

### Study design and subjects

This cross-sectional study was conducted among 16,000 formal and contractual employees of Esfahan Steel Company (ESCO) during 2014-2015 in Iran. Sample size in current epidemiological study was determined in order to achieve an accurate and reliable estimate of psychological problems prevalence. Considering the prevalence of psychological problems to be at least 0.1 (Andrea et al., 2004;[3] Sadeghirad et al., 2010[42]), type one error rate 0.05 and sampling error rate 0.01, sample size was found to be 3500. 

The inclusion criteria were as follows: work experience for at least one year, lacks of confirmed psychological disease, chronic physical diseases and enthusiasm in participation. Those participants who did not answer to a large fraction of questions (more than 10 % of the questionnaires' pages) were excluded from analysis. Participants were sampled in a multi-stage cluster sampling design along with stratified sampling based on managerial sections. Clusters were main seven managerial departments and their subsidiary sections and the strata were job categories of employees in ESCO. Employees in managerial and supervisory positions were considered as high-level white-collar employees (4.9 %) and office employees were considered as low-level white-collar employees (15.2 %). Blue collar employees were also classified as high-level (skilled workers; 16.4 %) and low-level (semi-skilled and non-professional workers; 63.5 %). Sample sizes in the clusters and strata were proportional to size. Due to random nature of sampling, as well as low number of female employees (n=800), in order to have sufficient women participants in our study, 260 females who agreed to participate in study were included using convenience sampling. Female employees all were working in official departmental jobs. The data gathering instruments were self-administrated questionnaires. The process of questionnaire administration and data collection was conducted at the company and study participants filled the self-administered questionnaires in their office. Research assistants guided participants regarding the possible ambiguities and also at the same time, the process was monitored rigorously by study coordinators during the study period. Data collection lasted 6 months. Data handling as well, including entering to computer and statistical software, quality assurance, rechecking the inclusion and exclusion criteria was done during 6 months. The study protocol was clarified for all participants and a signed written informed consent was obtained from them. Finally, 3,063 individuals (response rate 87 %) met proposed criteria; however, in current study data without missing were available for 3,056 individuals. Medical research ethics committee of the Isfahan University of Medical Sciences approved the study protocol (Research project number: 87115).

### Measurements

#### Psychological problem profile

Psychological problems profile, as a latent and integrated variable representing a comprehensive picture of mental health of study participants, was evaluated through combining three common psychological problems i.e. psychological distress, anxiety and depression. Psychological distress was measured by a self-administered 12-item GHQ-12 that participants answered having experienced a particular feeling or type of behaviour in a 4-point Likert scale as 'less than usual, no more than usual, fairly more than usual, or much more than usual' in the past few weeks (Goldberg et al., 1997[15]; Montazeri et al., 2003[30]). A participant could score between 0 and 12 points. GHQ-12 has been validated in Iranian population (Montazeri et al., 2003[30]).

Anxiety and depression were measured by Iranian validated version of the Hospital Anxiety and Depression Scale (HADS) (Montazeri et al., 2003[31]). HADS is a brief and useful questionnaire to assess the symptom intensity of anxiety disorders and depression. HADS consists of fourteen items, seven for anxiety and seven for depression. Items were measured on a 4-point Likert scale ranging from 0 (not present) to 3 (considerable). Maximum score for anxiety and depression is 21. Scores ≥ 8 on each subscale are considered as disorder while scores 0-7 are as normal (Montazeri et al., 2003[31]).

#### Stressful life events

Self-perceived frequency and intensity of stressful life events were assessed by Stressful Life Event questionnaire (SLE) (Roohafza et al., 2011[40]). The questionnaire consists of eleven domains including home life events (7 items: addiction of self or family member, divorce or separation, concern about addiction of a family member, quarrels with spouse, being accused, legal problems, troubles with children), financial problems (5 items: get in to debt, major financial problems, low income, taking on a mortgage, financial inflation), social relation (4 items: social discrimination, major social changes, social insecurity, concern about your future), personal conflict (5 items: lack of social support, cultural alienation, not having an intimate friend, failure in achieving the life goals, loneliness), job conflicts (4 items: quarrel with colleagues / boss, dealing with customers, increased working hours, improper working place and environment), educational concerns (4 items: failure in major examinations, participation major examinations, high educational expenses, educational problems of children), job security (5 items: job lay off, long lasting unemployment, concern about job future, high responsibility job, low salary), loss and separation (4 items: death of close family member, major disease of family members leading to hospitalization, death of parents, spouse or siblings, children's separation from family), sexual life (4 items: pregnancy, unwanted pregnancy, birth of a child, sexual relationship problems), daily life (2 items: air pollution and traffic, major changes in sleeping and eating habits), and health concerns (2 items: mild illness, major physical disease leading to hospitalization). All items were measured on a 6-point Likert scale (0: never, 1: very mild, 2: mild, 3: moderate, 4: severe, 5: very severe) and participants were asked about their life stressors at 6 months ago. More details about SLE questionnaire and its validity in Iranian population have been previously reported (Roohafza et al., 2011[40]).

### Covariates 

Association of psychological problems profile scores and stressful life events was adjusted for the impacts of some potential confounders including: demographic variables consist of sex, age (years), marital status (single, married), formal educational levels (< 6 years, 6 and 12 years and > 12 years) and household size.

Job related variables included shift work and job stress. Job stress was measured by a Persian validated version of the effort-reward imbalance questionnaire (F-ERIQ) (Yadegarfar et al., 2013[47]). F-ERIQ has 23 items similar as its English version (Siegrist, 1996[43]) in which 6 items are related to effort, 11 items regarding reward and 6 items for evaluating over-commitment. Sum of the respective scores for effort and reward and an adjusted ratio of the effort to reward were computed as the effort-reward imbalance (ERI) scale. Higher values of the ERI scale indicate a mismatch between effort and reward; in which participants with a higher effort/reward ratio score are more prone for experiencing job strain. The Cronbach's α coefficients of Persian version of ERI for effort, reward and over-commitment subscales were 0.61, 0.85 and 0.67, respectively (Yadegarfar et al., 2013[47]).

Lifestyle-related covariates in the current study included sleep duration (hour), ever smoking, BMI (kg/m^2^) and physical activity. Physical activity was assessed by the International Physical Activity Questionnaire (IPAQ) (IPAQ Research Committee, 2005[18]). The short form of World Health Organization's IPAQ contains items about intense activity, medium-intense activity and walking and sitting activities during the last seven days. Intensity of physical activity for each position is calculated based on the energy cost by metabolic equivalent (MET) in hour during the week. Summation of three obtained scores in IPAQ i.e. intense physical activity score, medium-intense physical activity score and walking and sitting activities score were considered as a total measurement for intensity of physical activity. IPAQ has been validated for an Iranian general population (Moghaddam et al., 2012[28]).

### Statistical analysis

Mean ± standard deviation (SD) was used for presenting quantitative variables and frequencies (percentages) for qualitative variables across quartiles of psychological problems profile. Analysis of variance and Chi-square test were used respectively for comparing quantitative and categorical variables across quartiles of psychological problems profile scores.

The Bayesian quantile structural equation model (quantile SEM) was applied for an integrative and comprehensive assessment of the associations of psychological problems profile scores and stressful life events. In measurement part of the quantile structural equation model, psychological problems profile as a latent variable was computed as a construct composed of depression, anxiety and psychological distress using confirmatory factor analysis and 11 stressful life events were integrated through explanatory factors analysis into two major domains i.e. socioeconomic and personal stressors (two new latent variables). In the structural part of the model, the relationship between quantiles of psychological problems profile scores as latent response and socioeconomic and personal stressors as latent predictors was evaluated. Potential confounders including demographic, job-related and lifestyle-related variables also were considered in the structural part as fixed covariates. Regression coefficients in the structural part of the fitted models and 95 % credible intervals (CI), were reported as measure of the associations. The associations were considered as statistically significant when the 95 % CIs did not include zero. Goodness of fit of the model was investigated by posterior predictive p-value (PP *p-*value) (Lee, 2007[24]; Meng, 1994[27]). A model can be considered plausible if its PP *p-*value is not far from 0.5. The quantile SEM was conducted by R Software (version 3.4) and model parameters were estimated using Bayesian approach. 

Also, conventional SEM was fitted and its results were compared with quantile SEM. Goodness of fit for the conventional SEM was assessed by Tucker-Lewis coefficient (TLI), comparative fit index (CFI) and root mean square error of approximation (RMSEA). RMSEA values less than 0.10 (Hooper et al., 2008[17]) and TLI and CFI values greater than 0.8 indicating an acceptable fitness (Browne et al., 1993[6]; Hair et al., 2013[16]).

## Results

Mean age of participants was 36.73 ± 7.30 years and 2798 (91.6 %) were male, 2,753 (90.1 %) were married, 62.30 % of employees had more than 12 years of educational level and 54.9 % were shift workers. Job stress score, based on ERI model, for 75 % of employees was more than 0.52 %; indicating majority of study participants are at high risk of job stress. 

Using confirmatory factor analysis, psychological problems profile was extracted as a latent construct by combining three observed mental health problems i.e. depression, anxiety and psychological distress (factor loadings have not been shown). The extracted factor explained 79.26 % of total variance. Then, factor scores were computed as intensity of psychological problems for participants so a higher value of psychological problems profile score indicates higher levels of psychological problems in an employee. 

Table 1(Tab. 1) presents the distribution of demographic, lifestyle and job-related characteristics of study participants across quartiles of psychological problems profile scores. Females, shift workers and smokers were more distributed in higher quartile of psychological problems profile scores (*p-*value<0.05). Mean ERI scale and sleep duration was significantly different across quartiles (*p-*value<0.05). 

**Figure 1:**
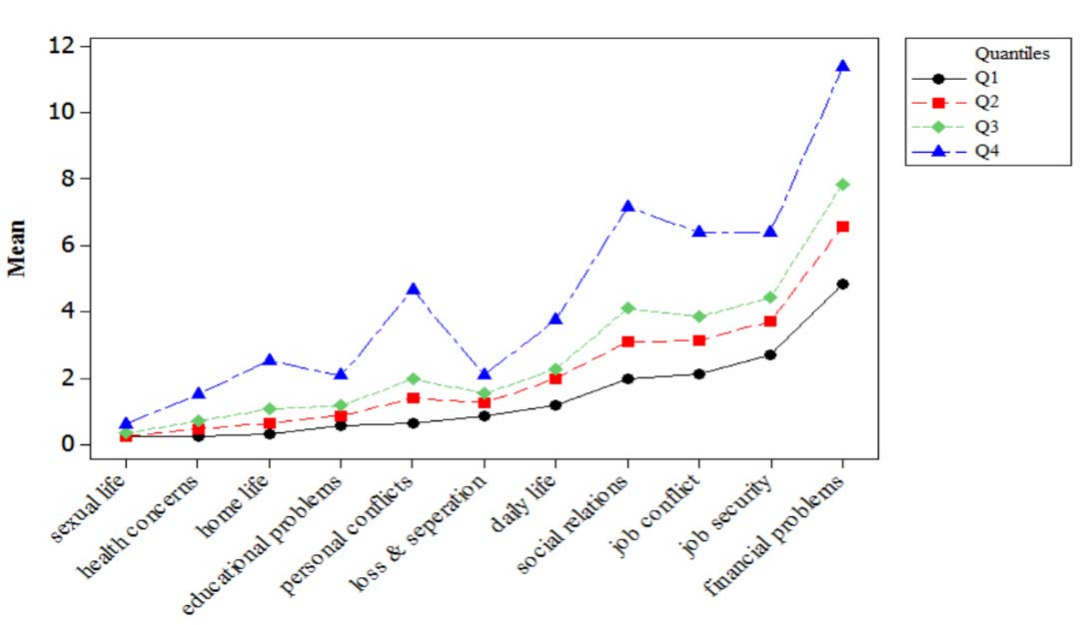
Mean of stressful life events across quartiles of psychological problems profile scores

Figure 1(Fig. 1) shows means of stressful life events in eleven domains across quartiles of psychological problems profile scores. As can be seen, sexual life has lowest mean and daily life and financial problems have highest means in all quartiles. Statistically significant differences were found between means of all stressors across quartiles (*p*-value<0.05). 

Applying exploratory factor analysis using correlation matrix, varimax rotation and scree plot on eleven life stressors domains resulted two factors explaining 45.63 % of total variance. The results are shown in Table 2(Tab. 2). First factor was labeled as "socioeconomics stressors" that more loaded by financial problems, social relations, personal conflicts, job conflicts, educational concerns, job security and daily life the second extracted factor was labeled as "personal stressors" more loaded by home life, loss and separation, sexual life and health concerns stressors.

To assess the association of psychological problems profile with stressful life events along with adjusting the impacts of potential confounders, a quantile structural equation model was fitted. Empirical distribution of psychological problems profile scores was notably skewed; so, quantile structural equation model as a robust approach against departure from normality was adopted.

**Figure 2:**
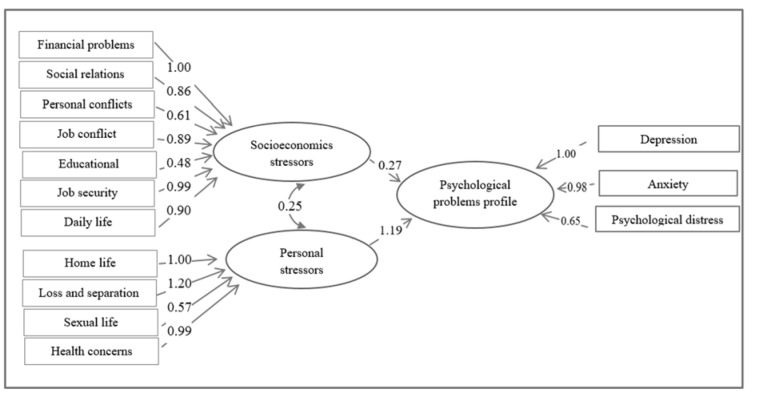
Psychological problems profile in association with stressful life events in the context of structural equation modeling approach

The framework of proposed structural equation model is shown in Figure 2(Fig. 2). In the measurement part of the model, latent variables (ovals) were linked to related observed variables (rectangles). In the structural part of the model, quantiles of latent response i.e. psychological problems profile were regressed on latent predictors i.e. socioeconomics and personal stressors.

Table 3(Tab. 3) shows the estimated coefficients of two latent stressors as predictors across five quantiles 0.05, 0.25, 0.50, 0.75, 0.95 of latent response variable i.e. psychological problems profile using quantile SEM. PP *p*-value as a goodness of fit measure is near 0.5 (ranged 0. 51-0.53), confirming an acceptable fitness.

Also, the results of fitting conventional linear SEM were reported in column “mean”; all fitting criteria (CFI ranged 0.859-0.935, TLI ranged 0.842-0.920, RMSEA ranged 0.051-0.061 through fitted models) confirmed goodness of the fitted models. 

In all models there were statistically significant positive association between both dimensions of stressful life events and psychological problem profile scores. Regression coefficients indicated stronger association between personal stressors and psychological problems profile scores across quantiles compared with socioeconomics stressors.

**Figure 3:**
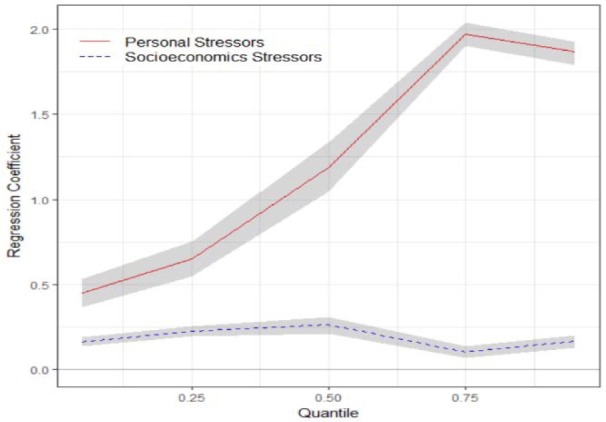
Estimated regression coefficients (solid lines and dash) and the 95 % credible intervals (grey area) of the association of two dimensions of stressful life events with quantiles of psychological problems profile scores

Figures 3(Fig. 3) depicts that the personal stressors dimension was positively associated with psychological problems profile scores and the association was stronger in higher quantiles. This means that the employees with higher levels of psychological problems scores more experienced higher levels of personal stressors. Socioeconomics stressors dimension also positively associated with psychological problems scores, but lower than personal stressors. The socioeconomic stressors showed nearly constant associations across the quantiles of psychological problems profile scores.

**Figure 4:**
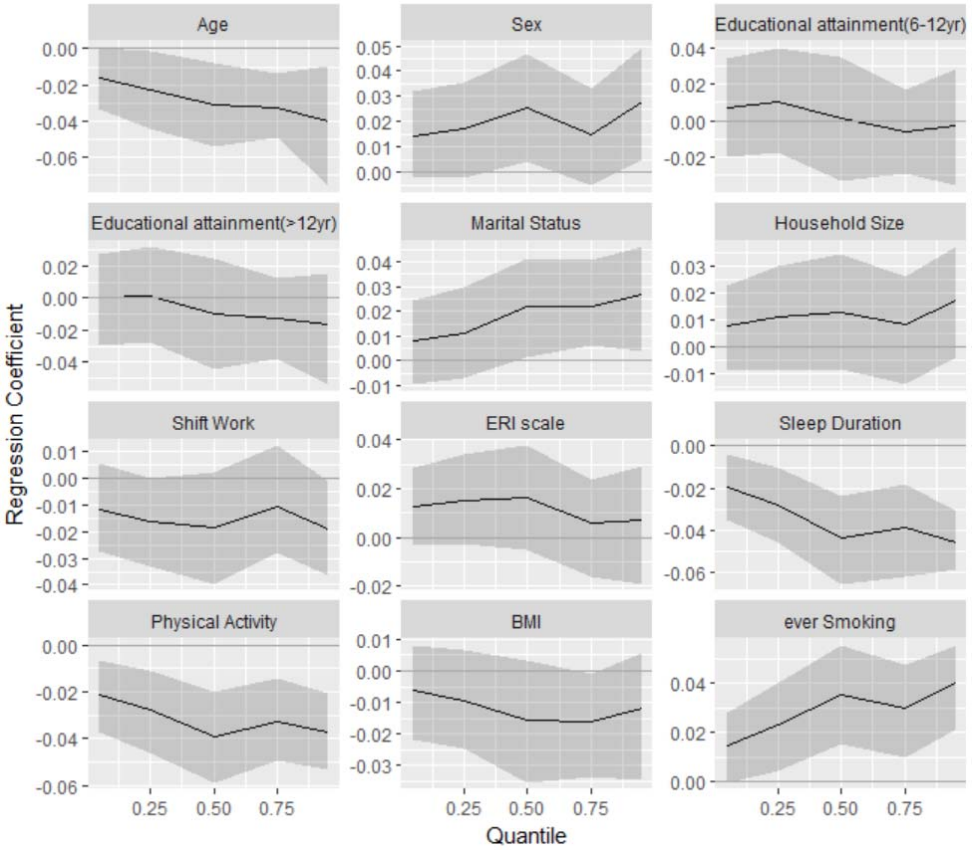
Estimated regression coefficients (solid lines) and the 95 % credible intervals for association of the confounding variables with quantiles of psychological problems profile scores (reference category for Sex: male, for marital status: married, for smoking: non-smokers and for educational attainment: less than 6 years)

Quantile regression coefficients for the association of confounding variables with psychological problems profile scores have been illustrated in Figure 4(Fig. 4). If 95 % credible interval for one variable does not cover the zero line it would indicate a statistical significant association.

Age had a significant inverse association with psychological problems profile score and the association was stronger in upper quantiles in which people with lower age had higher levels of psychological problems. Being female was significantly associated with increasing psychological problems profile scores constantly across quantiles. Marital status was significantly associated with psychological problems profile from median to the upper quantiles in which married people had lower psychological problems profile scores. Shift work and ERI scale as indicators of job strain did not show statistically significant association with psychological problems profile in presence of life stressors. Short sleep duration was significantly associated with more intensity of psychological problems and the association was stronger in higher quantiles of psychological problems profile scores. Physical activity showed a significant protective association with psychological problems; less physical activity was significantly associated with higher psychological problems profile scores. Smokers experienced higher significant psychological problems profile scores than non-smokers and the observed associations were stronger in upper quantiles (Figure 4(Fig. 4)).

## Discussion

In this study, we evaluated major domains of stressful life events and their association with psychological problems profile scores in a large sample of Iranian industrial manufacturing employees. A combined measure was obtained from three common psychological problems including depression, anxiety and psychological distress (i.e. psychological problems profile) and two dimensions were extracted from 11 stressful life events; i.e. personal stressors (home life, loss and separation, health concerns and sexual problems) and socioeconomics stressors (financial problems, social relations, job conflicts, job security, personal conflicts, daily life). 

Using quantile structural equation model, it was found that the employees with higher levels of psychological problems had experienced higher levels of personal stressors. Socioeconomics stressors was also positively associated with psychological problems profile scores but the association was almost uniform across quantiles of employees' psychological problems profile scores, it means that employees with different level psychological problems had experienced similar levels of socioeconomic stressors. Also, our study showed that the personal stressors had stronger association with psychological problems profile scores compared with socioeconomics stressors, these results are in line with previous studies and the theory behind Holmes and Rahe Stress Scale (Noone, 2017[34]). McLaughlin and Hatzenbuehler's study (2009[26]) indicated that stressful life events are longitudinally associated with increased anxiety, sensitivity and particularly family conflicts and health concerns. In a study among Canadian employees using structural equation modeling it was concluded that the work stressors composed of psychological demands, decision latitude, work social support and job insecurity had positive association with psychological distress, as well life stressors composed of chronic stressors and recent life events showed larger positive relationship (Cole et al., 2002[9]). In line with our results, in a study on employed men and women aged 30-60 it was found home stress was related to elevated depression and anxiety symptoms for both men and women, independent of job insecurity (Fan et al., 2015[11]). 

Among the addressed life event stressors in present study, financial and daily life had the highest and sexual problems and health concerns had the lowest intensity among studied population. Although, financial and daily stressors more perceived than sexual and home life stressors by employees, the results of quantile regression model showed that the personal stressors had more irritating impact on employees' psychological condition than socioeconomics stressors. This finding is consistent with Holmes and Rahe Stress Scale; in which death of a spouse or close family member, marital separation, personal injury or illness are top most stressful life events that might cause psychological and physical illness in adults (Noone, 2017[34]). A possible reason in this regard, is that the levels of perceived event importance differently influenced by individual 's mental and psychological conditions (Reiland and Clark, 2017[39]). 

In the present study, in line with results of previous studies, it was found also that the life stressors had independent association with psychological problems profile scores in the presence of job-related confounders and ERI scale. Clark and colleagues (2012[8]) and Phelan and colleagues (1991[38]) showed that occupational and domestic stress are related to major depression and depressive symptoms; and the relationships were independent of each other and persisted even after adjusting for the impacts of relevant sociodemographic and clinical confounders. 

In the present study, in line with previous literature, we observed that some potential confounding variables such as shorter sleep duration (Glozier et al., 2010[14]), low level of physical activity (Zschucke et al., 2013[48]) and smoking (Fluharty et al., 2017[13]) had significant association with higher scores of psychological problems profile. In this study, similar as previous studies, we observed that the married people and men had lower level of psychological problems scores (Bazazan et al., 2014[4]). In our study, increasing in age showed an inverse association with psychological problems intensity that it was in line with the results of studies by Akhtar-Danesh and Landeen (2007[1]); they found lower lifetime and 12-month's prevalence of depression in older age groups. However, the study of Rusli and colleagues (2008[41]) and a study conducted among general population in Iran using GHQ-28 questionnaire showed a direct association between mental disorders and age (Noorbala et al., 2004[35]). Conflicts in the findings may be attributed to different cultural, racial-ethnic backgrounds. 

Some strengths of the current study are: investigating of the association between a wide range of life stressors with psychological problems in the presence of many confounding demographic, lifestyle and job-related variables using a comprehensive statistical method in a large sample of industrial manufacturing employees in a developing country. Although, we could consider psychological problems as binary or multinomial variables and modeling them using a logistic regression, as it was done in previous studies, however quantile regression approach provided a more comprehensive evaluation about the association of mental health and life stressors. 

Limitations of the current study included cross-sectional analysis, in which cause and effect association could not be inferred. Another limitation is that the assessment of perceived frequency and intensity of life stressors may be explained by social preference or memory bias. Also, some potential confounders such as personality traits were not included in analyses, because personality traits, psychological problems and stressors are highly intercorrelated (Kendler et al., 2004[20]). 

In conclusion, life stressors particularly personal stressors are positively associated with higher scores of employees' psychological problems profile. Employees with more intensity of psychological problems perceive more life stressors confirming the importance of early detection of psychological problems. In prevention and treatment programs for psychological problems it is needed to consider diverse source of stress. Also, to ameliorate psychological health and improve productivity, educating suitable copying styles in order to adjust the adverse impacts of life stressors may be effective in occupational health interventions.

## Conflict of interest

The authors declare that there are no conflicts of interest.

## Acknowledgements

This project is based on PhD thesis in Biostatistics supported in part by Vice Chancellor for Research and Technology, Isfahan University of Medical Sciences (Study project number: 3941061). We are grateful to all volunteers who participated in this study. We would also like to acknowledge head of Esfahan Steel Company and staff of Cardiovascular Research Institute who helped us to conduct this study.

## Figures and Tables

**Table 1:**
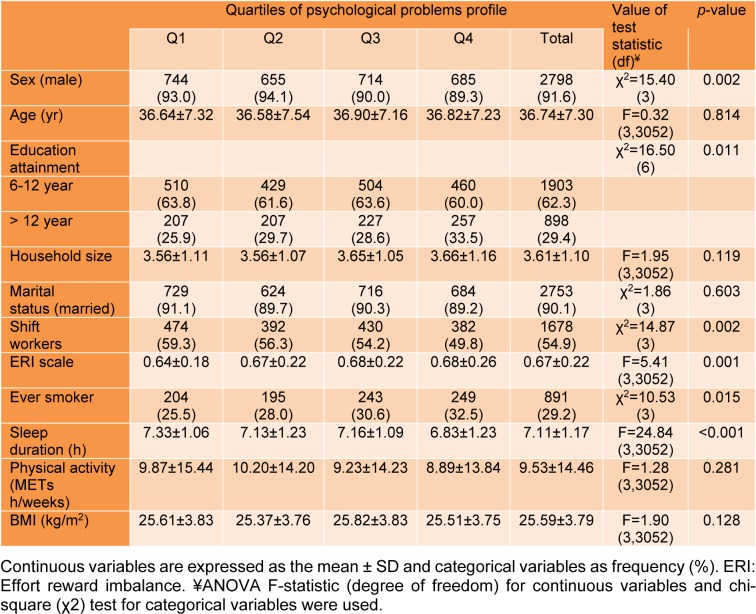
Demographic, job and lifestyle-related variables of total sample across quartiles (Q1-Q4) of psychological problems profile scores

**Table 2:**
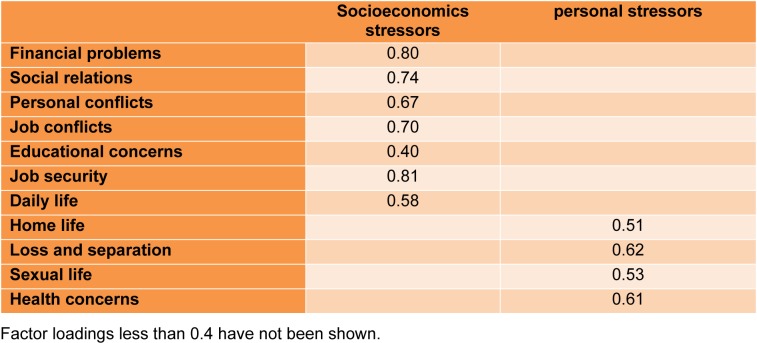
Factor loadings of two extracted factors from stressful life events

**Table 3:**
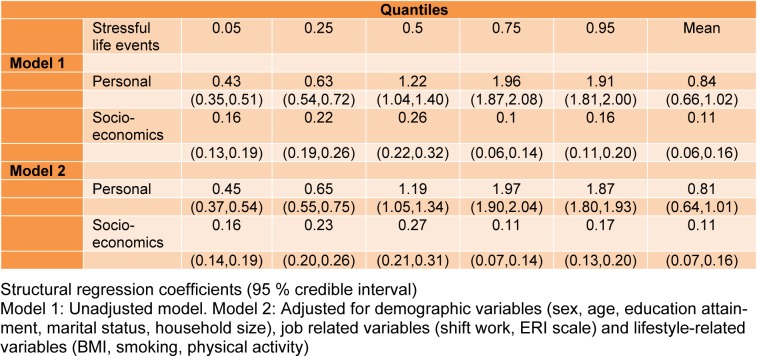
Bayesian estimated regression coefficients of the association of psychological problems profile and life stressors dimensions in the framework of quantiles structural equation model
